# Pamidronate-induced irreversible symptomatic hypocalcemia in a dog with hypercalcemia after glucocorticoid withdrawal: a case report

**DOI:** 10.1186/s12917-024-04030-x

**Published:** 2024-05-24

**Authors:** Ye-In Oh, Ju-Hyun An, Ga-Hyun Lim, Su-Min Park, Tae-Hee Kim, Kyoung-Won Seo, Hwa-Young Youn

**Affiliations:** 1 Department of Veterinary Internal Medicine, College of Veterinary Medicine & Institute for Veterinary Biomedical Science, Kyungpook National University, Daegu, 41566 Republic of Korea; 2 Department of Veterinary Emergency and Critical Care Medicine, Institute of Veterinary Science, College of Veterinary Medicine, Kangwon National University, Chuncheon-si, 24341 Republic of Korea; 3 Laboratory of Veterinary Internal Medicine, College of Veterinary Medicine and Research Institute for Veterinary Science, Seoul National University, Seoul, 08826 Republic of Korea

**Keywords:** Pamidronate, Hypocalcemia, Hypercalcemia, Dog, Glucocorticoid

## Abstract

**Background:**

Pamidronate is used for the treatment of hypercalcemia. However, a rare but potential adverse event of pamidronate treatment is hypocalcemia. This report describes an unusual case of severe, irreversible hypocalcemia after a single injection of pamidronate for the treatment of hypercalcemia due to glucocorticoid withdrawal in a dog.

**Case presentation:**

An 11-year-old castrated male Maltese dog presented with anorexia, vomiting, and diarrhea (day 0). The patient had calcinosis cutis throughout the body, calcification of intraabdominal organs, mild azotemia, and severe hypercalcemia. The severe calcification was attributed to long-term glucocorticoid administration, which was discontinued 1 month before presentation. Fluid therapy, diuretics, calcitonin, and a single intravenous injection of pamidronate were used for the treatment of hypercalcemia. On day 14, normocalcemia was achieved, but renal failure occurred. On day 20, severe and irreversible hypocalcemia occurred, and on day 42, the patient was euthanized at the owner’s request because of worsened hypocalcemia and renal failure.

**Conclusions:**

Although hypocalcemia is an extremely rare adverse event of bisphosphonate treatment, bisphosphonates like pamidronate can result in potentially life-threatening conditions according to the patient’s underlying conditions. Therefore, the patient’s condition should be closely monitored and any underlying conditions should be carefully evaluated before initiating the treatment for hypercalcemia using pamidronate.

**Supplementary Information:**

The online version contains supplementary material available at 10.1186/s12917-024-04030-x.

## Background

Serum calcium concentration is controlled by regulatory hormones including parathyroid hormone (PTH), calcitriol (1,25-dihydroxyvitamin D), 24,25-dihydroxyvitamin D, and calcitonin [1, 2]. In dogs, the most common causes of hypercalcemia are neoplasia, primary hyperparathyroidism, chronic kidney disease (CKD), and hypoadrenocorticism [2]. Hypercalcemia can be classified as parathyroid-dependent (primary hyperparathyroidism) and parathyroid-independent (normal parathyroid glands) [3]. In parathyroid-independent hypercalcemia, normal parathyroid glands suppress the production of PTH in a normal response to hypercalcemia, and PTH is typically undetectable or within the lower quartile of the reference range [3–5]. The causes of parathyroid-independent hypercalcemia include idiopathic hypercalcemia, malignancy, hypervitaminosis D, chronic inflammation, acute renal failure, and skeletal lesions [1, 2]. In general, when the serum ionized calcium (iCa) level exceeds 2.2 mmol/L, the patient becomes critically ill [2]. Treatments for hypercalcemia include fluid therapy and the administration of furosemide, prednisolone, calcitonin, and bisphosphonates [2].

Bisphosphonates bind to the mineral on the bone surface and inhibit the function of osteoclasts, thereby reducing bone resorption and consequently the serum calcium concentration [1, 6]. In dogs, nitrogen-containing bisphosphonates, such as alendronate, pamidronate, and zoledronate, are mainly used for treating hypercalcemia [7]. Nitrogen-containing bisphosphonates inhibit the mevalonic acid metabolic pathway at nanomolar concentrations, thereby interfering with protein prenylation required for normal intracellular signal transduction in the cytosol [7, 8]. This process is essential for bone resorption. In dogs, pamidronate is mainly used to control cancer-related bone pain; it is also used to treat hypercalcemia, but the incidence of adverse events remains unclear [9–16]. As in humans, the incidence of adverse events in dogs has been estimated to be low [6]. These adverse events include hypocalcemia, electrolyte imbalance, and renal failure, which are rare in both humans [17] and dogs [9, 10]. The acute-phase adverse events of pamidronate peak at 28–36 h after administration and generally self-limit within 2–3 days [18]. Intravenous bisphosphonates, such as pamidronate, are more likely to induce hypocalcemia than are oral bisphosphonates, and hypocalcemia usually occurs within a few days after administration. In humans, pamidronate-induced hypocalcemia mainly occurs in patients with preexisting hypovitaminosis D, hypoparathyroidism, and hypomagnesemia [19]. To prevent pamidronate-induced hypocalcemia, vitamin D and calcium should be supplemented starting 2 weeks before pamidronate administration [18]. Severe nephrotoxicity is another associated adverse event of intravenous pamidronate administration [17]. In humans, risk factors that can decrease renal function have been identified, including CKD, hypercalcemia, hypertension, older age, chemotherapeutic drugs, previous bisphosphonate treatment, and multiple myeloma [17]. However, few studies have evaluated this topic in veterinary medicine.

Herein, we report the unusual case of a dog that experienced severe and irreversible hypocalcemia after receiving pamidronate for the treatment of hypercalcemia due to glucocorticoid withdrawal and was subsequently diagnosed as having CKD. This case report aims to raise awareness about pamidronate-induced severe hypocalcemia as a rare but potentially life-threatening complication.

## Case presentation

### The patient information

**Fig. 1:**
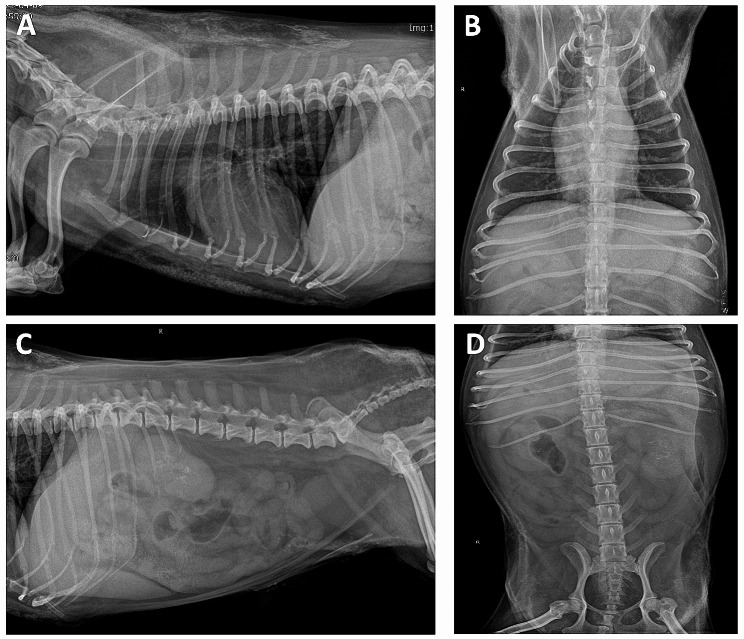
Radiographic image showing calcifications on the subcutis and spleen. **(A)** Lateral view of the thorax; **(B)** ventrodorsal view of the thorax; **(C)** lateral view of the abdomen, and **(D)** ventrodorsal view of the abdomen

**Fig. 2:**
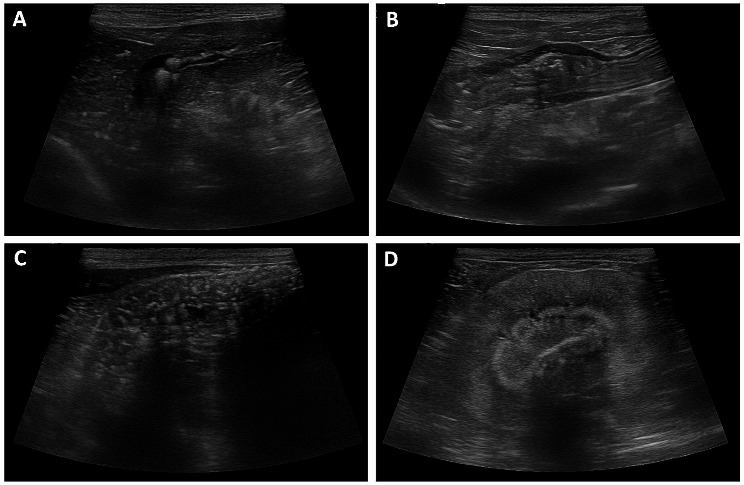
Abdominal ultrasonography showing multiple dystrophic calcifications on several organs. **(A)** Liver, **(B)** stomach, **(C)** spleen, and **(D)** left kidney

An 11-year-old castrated male Maltese dog weighing 4.2 kg presented with a 1-week history of partial anorexia, depression, tremor, vomiting, and diarrhea (day 0). The patient had severe calcinosis due to the long-term administration of oral glucocorticoids (prednisolone) for the treatment of recurrent immune-mediated hemolytic anemia (IMHA), but glucocorticoid administration was discontinued 1 month before presentation. This dog has had 3 episodes of IMHA in the last 18 months, each of which was started on prednisolone at 1–2 mg/kg bid with tapering at 1–4 week intervals, for a total of 10 months on prednisolone. A physical examination revealed lethargy, normal body condition score of 5/9, dehydration (7%), no enlarged lymph nodes and mass from head to perianal region, and calcinosis cutis throughout the skin. The rectal temperature was 39.1 °C. Blood analysis, including complete blood cell count, biochemistry, blood gas, and PTH levels, revealed neutrophilic leukocytosis (20,320/µL, reference range > 5200/µL) with a left shift, mild increased liver enzyme levels (ALT 126 U/L, reference range 5.8–83.3 U/L; ALP 206 U/L, reference range 0-97.9 U/L), mild azotemia (BUN 43.9 mg/dL, reference range 9.6–31.4 mg/dL; Cr 1.78 mg/dL, reference range 0.4–1.3 mg/dL), hypercalcemia (total Ca 15.7 mg/dL, reference range 9-11.9 mg/dL; ionized Ca 2.5 mmol/L, reference range 1.12–1.4 mmol/L, normal basal cortisol level (6.55 µg/dL, reference range 1–6 µg/dL, and low PTH level (2 pg/mL, reference range 20–130 pg/mL) (Table 1). Thoracic and abdominal radiography revealed generalized calcification on the subcutis and spleen (Fig. 1). Abdominal ultrasonography revealed multiple dystrophic mineralizations, including on the gall bladder wall, liver, stomach, spleen, and kidney (Fig. 2). The underlying diseases were gastroenteritis, chronic pancreatitis, and CKD, with International Renal Interest Society (IRIS) stage 2; however, anemia was absent. The patient was on mycophenolate mofetil 3 mg/kg bid (CellCept, Roche Korea, Seoul, Republic of Korea), cyclosporin 5 mg/kg bid (Sandimmun, Novartis, Basel, Switzerland), famotidine 0.5 mg/kg bid (Daehwa Pharmaceutical, Seoul, Republic of Korea), silymarin 20 mg/kg bid (Sinil Pharmaceutical, Seoul, Republic of Korea), ursodeoxycholic acid 20 mg/kg bid (Urusa, Daewoong Pharmaceutical, Seoul, Republic of Korea), and Zentonil® 100 mg/dog sid (Vetoquinol, Canada).

**Table 1:** Laboratory test results during the treatment course

Item (reference range)	During hypercalcemia (hospitalization)					During hypocalcemia (outpatient)				
Day	0	1	3	8	10	14	20	27	35	42
WBC (> 5200/µL)	20,320	26,790	16,840	21,490	20,290	-	42,000	13,970	26,100	32,550
RBC (570–880 × 10^4^/µL)	623	559	592	605	566	-	534	516	485	431
HCT (37.1–57%)	39.3	33	37.9	35.8	34.3	-	33.3	32.3	29.3	26.4
Total Ca (9-11.9 mg/dL)	15.7	> 16	14.5	> 16	13	13.9	5.9	7.1	5.4	3.5
Ionized Ca (1.12–1.4 mmo/L)	2.5	2.23	1.98	2.14	2.01	1.1	0.83	0.85	0.72	0.62
BUN (9.6–31.4 mg/dL)	43.9	50	75.3	38	44.8	104.3	148.6	104.9	130.3	128.6
Cr (0.4–1.3 mg/dL)	1.78	1.9	3.12	2.6	2.54	5.18	6.81	5.26	5.76	5.18
P (2.3–6.3 mg/dL)	5.4	8.2	7.9	11.7	10.2	10	10.5	11.5	10.3	9.5
PTH (20–130 pg/mL)	2	-	-	-	-	-	-	-	-	-
Cortisol (1–6 µg/dL)	6.55	-	-	-	-	-	-	-	-	-

WBC, white blood cell; RBC, red blood cell; HCT, hematocrit; BUN, blood urea nitrogen; Cr, creatinine; P, phosphorus; PTH, parathyroid hormone

### Diagnostic assessment

Severe hypercalcemia was diagnosed as the cause of the clinical signs and iCa levels were measured serially with a blood gas analyzer (GEM PREMIER 5000, Instrumentation laboratory, Italy) for monitoring during hypercalcemia treatment. The patient was treated using fluid therapy (0.9% normal saline), furosemide (Dailix, Sinil Pharmaceutical, Republic of Korea), pamidronate (2 mg/kg in 100 ml 0.9% normal saline) for 2 h constant rate infusion (CRI) (Panorin, Hanlim Pharmaceutical, Seoul, Republic of Korea), and sodium bicarbonate 4 mEq/kg slow intravenous (IV) administration (Daewon Pharmaceutical, Seoul, Republic of Korea). Five hours later, systemic hypertension occurred (systemic blood pressure > 200 mmHg), and monitoring was continued using the Doppler method after administering hydralazine 0.5 mg/kg IV (Samjin Pharmaceutical, Seoul, Republic of Korea) and nitroprusside 0.5 µg/kg/min CRI (Nitropress, Pfizer, USA). Intermittent tremors occurred during treatment. On day 1, iCa level decreased to 1.75 mmol/L (reference range, 1.12–1.4 mmol/L) in the morning, and then increased to 2.24 mmol/L in the night; however, as systemic hypertension continued, amlodipine 0.2 mg/kg bid (Myungmoon Pharmaceutical, Seoul, Republic of Korea) was added. As azotemia worsened and hyperphosphatemia developed, aluminum hydroxide 30 mg/kg bid (Gelusam, Samnam Pharmaceutical, Seoul, Republic of Korea), lanthanum carbonate 30 mg/kg bid (Fosrenol, Shire, USA), and sevelamer 30 mg/kg bid (Invela, SK Chemicals, Seoul, Republic of Korea) were started. On day 3, calcitonin 4 IU/kg sc bid was added to reduce the iCa level (1.98 mmol/L, reference range, 1.12–1.4 mmol/L). Nitroprusside treatment was discontinued to prevent cyanide toxicity. On day 7, furosemide was discontinued because azotemia and hyperphosphatemia did not improve. On day 8, after discontinuing furosemide, hypercalcemia slightly worsened, and hence, the calcitonin dose was increased to 6 IU/kg tid (Miacalcic, Novartis). On day 10, hypercalcemia still did not improve, but the patient was discharged at the owner’s request. At discharge, the patient was prescribed oral administration of cyclosporin 5 mg/kg bid, amlodipine 0.4 mg/kg bid, famotidine 1 mg/kg bid, maropitant 1 mg/kg sid (Cerenia, Zoetis, USA), tramadol 4 mg/kg bid (Shinpoong Pharmaceutical, Seoul, Republic of Korea), silymarin 20 mg/kg bid, ursodeoxycholic acid 20 mg/kg bid, Zentonil® 100 mg/dog sid, aluminum hydroxide 30 mg/kg bid, lanthanum carbonate 30 mg/kg bid, and sevelamer 30 mg/kg bid. On day 14, the patient still had partial anorexia and depression, but had normocalcemia and slightly improved calcinosis cutis. However, azotemia worsened and CKD reached IRIS stage 4. The owner still refused critical care and wanted home care using subcutaneous fluid therapy. IMHA did not recur, and hence, the cyclosporin dose was reduced to 2 mg/kg bid. Moreover, as normal blood pressure was maintained, the amlodipine dose was reduced to 0.2 mg/kg bid. The other treatments remained unchanged. On day 20, the patient showed persistent anorexia and lethargy, without other symptoms. Calcinosis cutis had improved more than 5 days before, but hypocalcemia occurred (0.83 mmol/L, reference range, 1.12–1.4 mmol/L). The owner refused any additional treatment but wanted to maintain monitoring. Therefore, calcium supplementation was not initiated. Blood pressure remained normal, and hence, the amlodipine dose was reduced to 0.1 mg/kg bid. On day 27, the clinical signs were similar, and the iCa level (0.85 mmol/L, reference range, 1.12–1.4 mmol/L)was still low. However, azotemia improved slightly and the blood pressure remained normal; hence, amlodipine was discontinued. On day 42, the patient presented with lateral recumbency and labored breathing. Non-regenerative anemia (PCV 26.4%, reference rage 37.1–57%; reticulocyte count 10/µL, reference range 0–60/µL; MCV 60.3 fl., reference range 58.8–71.2 fl.; MCH 20.4 pg, reference range 20.5–24.2 pg; MCHC 33.8 g/dl, reference range 31–36.2 g/dl) due to CKD had aggravated, and azotemia had worsened. The iCa level (0.62 mmol/L, reference range, 1.12–1.4 mmol/L) decreased further, and hence, the jaw tone increased and forelimb stiffness was observed. Although 10% Ca gluconate 1.2–1.5 ml/kg/h CRI (Daihan Pharmaceutical, Seoul, Republic of Korea) was performed, cardiopulmonary arrest occurred. We performed cardiopulmonary resuscitation, and the patient was resuscitated, but vital signs remained unstable, and the mental status remained unclear with no obvious improvement. Finally, euthanasia was performed at the owner’s request. Propofol 0.6 mg/kg iv (Provive 1%, Pharmbio Korea Inc., Republic of Korea) and T 61 ad us. vet. 0.3 ml/kg iv (Korea MSD animal health, Republic of Korea) were used for euthanasia.

## Discussion and conclusions

In this case, the patient developed iatrogenic Cushing’s syndrome during exogenous glucocorticoid therapy for IMHA, and it was controlled by tapering the glucocorticoid dose. After 3 weeks, the patient developed anorexia, which was later diagnosed as being due to symptomatic hypercalcemia. Pamidronate was administered once and other supportive care was provided to treat hypercalcemia. Nevertheless, the patient subsequently developed severe and irreversible symptomatic hypocalcemia and CKD, which led to death.

The hypercalcemia developed within 30 days of glucocorticoid withdrawal. This patient showed increased total calcium, iCa, and phosphorus levels and decreased PTH levels, and was diagnosed as having parathyroid-independent hypercalcemia because of the lower-than-normal PTH level. The typical causes of hypercalcemia are adrenal insufficiency, hypervitaminosis A, aluminum exposure, and toxicity (cholecalciferol, calcitriol, or calcipotriene) [2]. However, based on the history taking and test results, we could exclude all causes other than adrenal insufficiency. The cause of hypercalcemia can be diagnosed using blood tests including those for serum 25-hydroxyvitamin D, 24,25(OH)2-vitamin D, calcitriol, and PTH-related protein concentrations [3]. Unfortunately, in this case, the owner refused all tests other than the PTH test; hence, a definitive diagnosis was impossible. Nevertheless, the cause of hypercalcemia was presumed to be hormone imbalance due to glucocorticoid withdrawal. The patient had shown clinical characteristics such as polyuria/polydipsia, polyphagia, panting, distended abdomen, calcinosis cutis, and increased liver enzyme levels (alanine aminotransferase, aspartate aminotransferase, alkaline phosphatase, and γ-glutamyltransferase) during glucocorticoid withdrawal. Therefore, the tentative diagnosis was iatrogenic Cushing’s syndrome. The hypercortisolemia may have occurred because of long-term glucocorticoid use. The causes of hypercalcemia related to glucocorticoid withdrawal in this patient could be the following similar but non-identical conditions: (1) glucocorticoid-induced adrenal insufficiency; (2) glucocorticoid withdrawal syndrome; and (3) possible movement of calcium accumulated in the skin and organs into the bloodstream and subsequent increase in concentration.

Although the mechanism underlying hypercalcemia due to glucocorticoid withdrawal remains unclear, hypercalcemia may occur because of increased bone resorption and decreased renal excretion of calcium due to adrenal insufficiency [20–22]. The diagnosis and treatment of glucocorticoid-induced adrenal insufficiency and glucocorticoid withdrawal syndrome have been established in humans [23]. Both these conditions could be attributed to glucocorticoid withdrawal after long-term use. However, adrenal insufficiency poses a risk of adrenal crisis, whereas glucocorticoid withdrawal syndrome does not. Since patients with glucocorticoid withdrawal syndrome have normal hypothalamic-pituitary adrenal function, their basal cortisol levels are normal. Since this patient developed hypercalcemia and had no stress leukogram after long-term glucocorticoid use, the patient could have a condition similar to glucocorticoid-induced adrenal insufficiency. In this patient, when hypercalcemia occurred, no typical low Na: K ratio was observed. This suggested a possibility of glucocorticoid-deficient secondary adrenal insufficiency due to glucocorticoid administration. Although the adrenocorticotrophic hormone stimulation test was not performed, the basal cortisol level was within the reference range. Moreover, the diagnosis of typical adrenal insufficiency is difficult. In a human patient with Cushing’s syndrome, adrenal insufficiency developed after adrenalectomy, followed by secondary hypercalcemia [21]. That patient initially presented with anorexia, low adrenocorticotrophic hormone levels, and normal serum cortisol levels, but subsequently developed hypercalcemia. Since our patient visited the hospital only 1 week after symptom onset and was diagnosed as having hypercalcemia, the serum cortisol level may have been normal at this time, as in the human case above.

Another cause of hypercalcemia is multiple calcifications resulting from long-term glucocorticoid use. Although it occurs very rarely and the cause is unknown, cases of hypercalcemia due to glucocorticoid withdrawal have been reported in a dog [24] and a human [25]. Chronic prednisolone use for 6 months resulted in iatrogenic Cushing’s syndrome and severe calcinosis cutis throughout the body of that dog [24]. Although glucocorticoid discontinuation resulted in the resolution of calcinosis cutis, the dog developed marked hypercalcemia, presumably because calcium eluted from the calcified skin into the bloodstream. Since our patient also had a similar pathologic process, the cause of hypercalcemia could be similar.

In humans receiving systemic administration of glucocorticoids, factors that increase the risk of glucocorticoid withdrawal-induced adrenal insufficiency include daily administration for > 2–4 weeks, multiple daily split doses, and bedtime administration, and the factors that reduce the risk include alternate-day administration and pulse systemic therapy [22]. Nevertheless, clinical data on the protocols to reduce the glucocorticoid dose and thereby reduce these adverse events remain inadequate in both humans and animals. In humans, depending on the dose of glucocorticoid administered, a protocol of reducing the dose by 10–20% every 1–2 weeks and a protocol of reducing the dose by 20, 10, 5, 2.5 mg every 1–2 weeks have been suggested [26]. Nevertheless, studies on how to safely and effectively reduce the use of glucocorticoids over a long time in animals are currently lacking.

Our patient received fluid therapy, furosemide, calcitonin, and pamidronate to treat hypercalcemia, but developed very severe hypocalcemia. Considering the patient’s course and treatment characteristics, the main cause of hypocalcemia was pamidronate administration. Fluid therapy and furosemide do not cause persistent and severe hypocalcemia, and the calcium concentration continued to decrease even after the discontinuation of these treatments. Although calcitonin is fast-acting, it can induce only a moderate decrease in calcium concentration, and resistance to calcitonin can occur if used for several days [27–29].

Since an IV injection of pamidronate has a subacute effect onset, iCa levels begin to decrease within 72 h after administration [2]. One dog with cancer-related hypercalcemia developed hypocalcemia on receiving nine consecutive treatments of single-agent pamidronate, but although the clinical symptoms improved, the calcium levels did not normalize [9]. In three dogs with hypercalcemia secondary to an apocrine gland anal sac adenocarcinoma, symptomatic hypocalcemia developed after the injection of pamidronate prior to mass removal [30, 31]. The main cause of hypocalcemia in these dogs was that PTH-related protein was secreted by the tumor; in the presence of hypercalcemia, calcium concentration was lowered by removing the cancer, and pamidronate aggravated it. All these dogs achieved normocalcemia while receiving symptomatic treatment for hypocalcemia. In our patient, however, severe hypocalcemia occurred after the administration of only one dose of pamidronate. Another study on 95 dogs with cancer that were administered zoledronate, which is 100 times more potent than pamidronate, reported that only 9 dogs had hypercalcemia, and no hypocalcemia was observed after zoledronate administration [32]. In a human patient with hypercalcemia due to malignancy [33], the administration of one dose of pamidronate resulted in severe, irreversible hypocalcemia and death similar to our patient. That patient’s serum PTH and vitamin D concentrations were lower than the reference range. The risk factors for severe bisphosphonate-induced hypocalcemia are well known in humans, and include subclinical parathyroid gland function, vitamin D deficiency, impaired renal function, malabsorption syndrome, malnutrition, and severe hypomagnesemia. In our patient, PTH was not available at the time of the hypocalcemia, which is unfortunate. In this case, a PTH test to check parathyroids gland function would have been more helpful in differentiating the cause.

Vitamin D deficiency and CKD could be the factors contributing to the development of severe, irreversible hypocalcemia in the present case. Unfortunately, although we could not measure the serum vitamin D concentration in the current patient, we think that the vitamin D concentration decreased because of long-term glucocorticoid use and CKD, which were the underlying conditions in this patient. In canine CKD, as the IRIS stage increases, the vitamin D concentration tends to decrease, and in particular, in stages 3 and 4, the concentration is significantly lower than the reference range [34, 35]. In the present study, the patient had CKD with IRIS stage 2 at the time of the first diagnosis of hypercalcemia, but CKD worsened to IRIS stage 3 after 3 days.

A correlation between glucocorticoid use and serum 25-hydroxyvitamin D levels has been established in humans [36]. When glucocorticoids were used, the possibility of lower serum levels of 25-hydroxyvitamin D was half as likely compared to when glucocorticoids were not used. Glucocorticoids decrease intestinal calcium absorption, increase urinary excretion of calcium, and enhance bone resorption [37, 38]. Similarly, in dogs with hypercortisolism, the serum 25-(OH)D concentration was significantly lower than that in normal dogs [39]. Further studies are warranted to establish a clear standard, and in patients at risk, glucocorticoid use should be reduced and serum calcium concentration should be closely monitored. In humans, vitamin D concentrations are measured before the administration of bisphosphonates and supplemented to reduce the risk of hypocalcemia [19]. Therefore, the vitamin D concentration should be measured even in animals before initiating treatment with bisphosphonates. In this patient, hypocalcemia was fatal, and both hypocalcemia and CKD were presumed to be the major causes of death.

The risk of nephrotoxicity increases with higher doses, shorter administration times, and frequent administration of IV bisphosphonates, such as pamidronate and zoledronate [40]. This is rare with oral bisphosphonates. Cases of azotemia following the administration of pamidronate are rare in dogs [9, 41]. In previous studies [9, 41], a total of 34 dogs were injected with pamidronate and two of them developed azotemia after injection of pamidronate (diluted in a total volume of 250 mL 0.9% sodium chloride and administered as a 2-hour CRI). They had adenocarcinoma of anal sac and hypercalcemia in common. It is possible that azotemia occurred as a side effect of pamidronate due to hypercalcemia resulting in renal injury. Moreover, zoledronate is more likely than pamidronate to cause renal tubule injury [42]. When zoledronate was used in 95 dogs with cancers, mild azotemia developed in 20 dogs and moderate azotemia developed in only 1 dog [32]. In these dogs, zoledronate did not cause azotemia. Nonsteroidal anti-inflammatory drugs, renal neoplasia, postrenal obstruction, and prerenal azotemia were the causes of azotemia. In the present study, furosemide and calcitonin were also used in addition to pamidronate, and as the underlying CKD progressed rapidly with the use of several drugs that were nephrotoxic, CKD with IRIS stage 4 appeared to have a direct impact on the patient’s death together with hypocalcemia. Therefore, close monitoring of renal function should be performed in patients receiving pamidronate. In humans, if creatinine clearance is less than 30 mL/min, pamidronate administration is a contraindication [43, 44]. Although creatinine clearance is an important factor in determining the administration of pamidronate in humans, there is no such guideline for pamidronate administration in animals because measuring creatinine clearance in animals is difficult.

In summary, the dog had iatrogenic Cushing’s syndrome and developed severe hypercalcemia after glucocorticoid withdrawal. The use of pamidronate for the treatment of hypercalcemia resulted in hypocalcemia and rapid worsening of CKD. Although hypocalcemia is a rare side effect of bisphosphonate treatment, pamidronate can cause potentially life-threatening conditions. Therefore, a complete diagnostic approach to the cause of hypercalcemia is necessary before using pamidronate for the treatment of hypercalcemia, and the use of pamidronate should be carefully considered, especially in dogs that have undergone glucocorticoid withdrawal, and the appropriate treatment of hypercalcemia should be determined based on the underlying condition.

### Electronic supplementary material

Below is the link to the electronic supplementary material.

Supplementary Material 1

## Data Availability

The original contributions presented in the study are included in the article, and further inquiries can be directed to the corresponding author.

## Abbreviations

PTH: Parathyroid hormone
CKD: Chronic kidney disease
iCa: Ionized calcium
IMHA: Immune-mediated hemolytic anemia
IRIS: International Renal Interest Society
CRI: Constant rate infusion
