# Supplementary figures and images for: Pamidronate-induced irreversible symptomatic hypocalcemia in a dog with hypercalcemia after glucocorticoid withdrawal: a case report
Source: BMC Vet Res. 2024 May 24;20:227. doi: 10.1186/s12917-024-04030-x (PMC11127328; doi:10.1186/s12917-024-04030-x)

Supplementary file


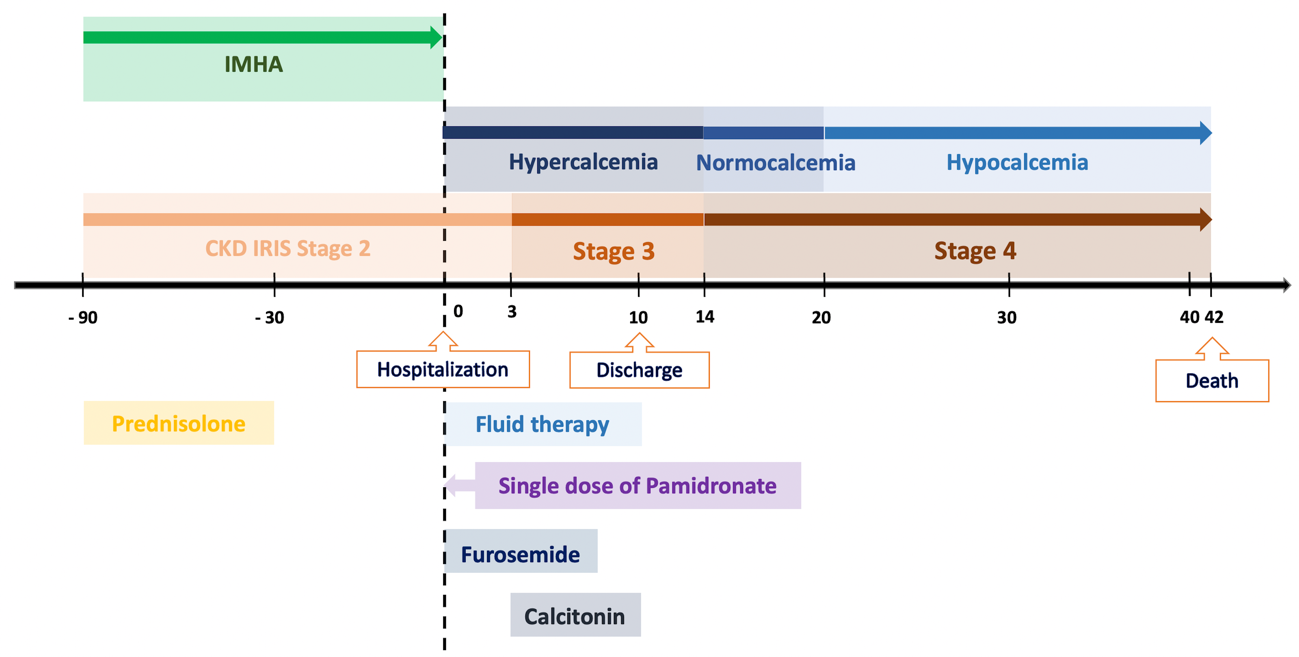


**Supplementary figure**

Patient’s timeline in this case report.

Supplement: Supplementary file 1 — Supplementary Material 1 [file 12917_2024_4030_MOESM1_ESM.docx]
